# Electromagnetic energy (670 nm) stimulates vasodilation through activation of the large conductance potassium channel (BK_Ca_)

**DOI:** 10.1371/journal.pone.0257896

**Published:** 2021-10-05

**Authors:** Debebe Gebremendhin, Brian Lindemer, Dorothee Weihrauch, David R. Harder, Nicole L. Lohr

**Affiliations:** 1 Department of Physiology, Medical College of Wisconsin, Milwaukee, WI, United States of America; 2 Cardiovascular Center, Medical College of Wisconsin, Milwaukee, WI, United States of America; 3 Division of Cardiovascular Medicine, Department of Medicine, Medical College of Wisconsin, Milwaukee, WI, United States of America; 4 Clement J Zablocki VA Medical Center, Milwaukee, WI, United States of America; 5 Department of Anesthesiology, Medical College of Wisconsin, Milwaukee, WI, United States of America; Cinvestav-IPN, MEXICO

## Abstract

**Introduction:**

Peripheral artery disease (PAD) is a highly morbid condition in which impaired blood flow to the limbs leads to pain and tissue loss. Previously we identified 670 nm electromagnetic energy (R/NIR) to increase nitric oxide levels in cells and tissue. NO elicits relaxation of smooth muscle (SMC) by stimulating potassium efflux and membrane hyperpolarization. The actions of energy on ion channel activity have yet to be explored. Here we hypothesized R/NIR stimulates vasodilation through activation of potassium channels in SMC.

**Methods:**

Femoral arteries or facial arteries from C57Bl/6 and Slo1^-/-^ mice were isolated, pressurized to 60 mmHg, pre-constricted with U46619, and irradiated twice with energy R/NIR (10 mW/cm^2^ for 5 min) with a 10 min dark period between irradiations. Single-channel K^+^ currents were recorded at room temperature from cell-attached and excised inside-out membrane patches of freshly isolated mouse femoral arterial muscle cells using the patch-clamp technique.

**Results:**

R/NIR stimulated vasodilation requires functional activation of the large conductance potassium channels. There is a voltage dependent outward current in SMC with light stimulation, which is due to increases in the open state probability of channel opening. R/NIR modulation of channel opening is eliminated pharmacologically (paxilline) and genetically (BK_ca_ α subunit knockout). There is no direct action of light to modulate channel activity as excised patches did not increase the open state probability of channel opening.

**Conclusion:**

R/NIR vasodilation requires indirect activation of the BK_ca_ channel.

## Introduction

Peripheral artery disease is a complex vascular pathology characterized by flow limiting obstruction of the peripheral conduit vessels and microvascular dysfunction [1]. Despite limited treatment modalities designed to alleviate conduit vessel obstruction and reduce risk factors associated with endothelial dysfunction (e.g., lipid lowering, plasma glucose levels, blood pressure), the morbidity and mortality associated with PAD remains very high [2,3]. Critical limb ischemia is the most severe form of PAD and often leads to limb amputation and poor quality of life [4]. Effective treatments to improve limb perfusion are vital to prevent amputation, however these modalities heavily favor invasive surgical and percutaneous treatments which have significant morbidity [5]. Moreover, the limited efficacy of surgical intervention in vascular beds distal to the popliteal artery and the high procedural costs should necessitate the development of non-invasive therapies [6].

A potential therapeutic method is the application of light energy to the skin. Numerous reports have associated light treatment with improved wound healing [7–10]. In animal studies, polychromatic visible light stimulates vasodilation [11–13]. Pharmacological inhibition of vessel segments stimulated with polychromatic light or high intensity red light has suggested multiple mechanisms for vasodilation including nitric oxide and the small and intermediate conductance potassium channels [13]. Interestingly, the vasodilation stimulated under these conditions has been endothelial independent.

Previously, our laboratory identified energy in the visible red to near infrared spectrum (R/NIR, 670 nm wavelength) could produce nitric oxide within the blood and myocardium from sources such as iron nitrosyl-hemoglobin and nitrosyl-myoglobin [14]. The NO produced from these moieties is significant, as it is cardioprotective in acute ischemia and stimulates angiogenesis [14,15]. The mechanism of NO generation by R/NIR is independent of functional nitric oxide synthases, which increases its clinical relevance since many patients with PAD have impaired NOS function. Recently, we have shown R/NIR to stimulate vasodilation of physiologically pressurized external carotid vessels in mice through a NO and endothelial dependent manner, which differs from previously reported light stimulated vasodilation studies where vasodilation required inhibition of the small and intermediate potassium channels [13,16,17]. Unlike these studies which rely on pharmacological inhibitors of the NO pathway, we sought to characterize the effect of R/NIR on smooth muscle cell ion channel activity. We demonstrate R/NIR increases the open state probability of the large conductance potassium channel.

## Materials and methods

All experimental procedures and protocols used in this investigation were reviewed and approved by the Animal Care and Use Committee of the Medical College of Wisconsin. Furthermore, all conformed to the *Guiding Principles in the Care and Use of Animals* of the American Physiologic Society and were in accordance with the *Guide for the Care and Use of Laboratory Animals*.

### R/NIR source

A 670 nm light emitting diode source was utilized for all pressure myography experiments and patch clamping experiments (NIR Technologies, Waukesha, WI). The patch clamping experiments were performed with a fiber optic cable attached to the R/NIR light source. Power output was measured with an irradiance meter (X97, GigaHertz-Optik). The light sources were placed 2.5 cm from their target in all pressure myography experiments. In vitro, the tip of the fiber optic cable was positioned approximately 5 cm above the bath.

### Pressure myography

Segments of facial arteries (160–260 μm ID) or femoral arteries (380–460 μm ID) from C57Bl6/J mice were transferred to a water-jacketed perfusion chamber and cannulated with two glass micropipettes (tip diameter ≈30 μm) at their in-situ length. The arteries were bathed in the PSS-equilibrated solution maintained at pH 7.4 and 37°C. The micropipettes were connected to a reservoir filled with physiological saline solution and the arteries were pressurized to 60 mmHg. The internal diameter of the arteries was measured with a video system composed of a stereomicroscope (Olympus CK 40), a charge-coupled device camera (Panasonic GP-MF 602), a video monitor (Panasonic WV-BM 1410), and a video measuring apparatus (Boeckeler VIA-100). All experiments were performed with the endothelium intact.

After a 1-hour equilibration period, the arteries were pre-constricted by ∼50% of their resting diameter with a thromboxane A_2_ analog, U-46619. To test the stability of U46619 we measured vessel diameters in a separate group up to 45 min after steady state. Diameters did not change during this test period. The vessels were placed in the dark and once steady-state contraction was obtained; they were treated with 10 mW/cm^2^ of 670 nm light for 5 minutes. The vessels were then allowed to recover in the dark for 10 minutes and then the R/NIR light was reapplied for another 5 minutes. The vessels were then allowed to recover in the dark for another 5 minutes. Passive vasodilator responses to papaverine (10^−4^ M) were determined at the end of each experiment. Vascular responses are expressed as percent maximal relaxation of the U-46619-induced constriction, with 100% representing the passive increase in diameter with papaverine.

### Dissociation of mouse femoral arterial smooth muscle cells

Femoral arteries were isolated from wild type mouse (8–10 weeks of age) anesthetized with inhalational 2% isoflurane. Isolated femoral arteries were placed in low-calcium dissociation solution composed of (in mM): NaCl 134, KCl 5.4, MgSO_4_ 1.2, KH_2_PO_4_ 0.24, CaCl_2_ 0.01, glucose 11 and HEPES 10 (pH adjusted to 7.4 with NaOH). The femoral arterial segments were transferred into 1 mg/ml bovine serum albumin (Sigma) in low-calcium dissociation solution and kept for 10 minutes at room temperature. The arterial segments were then transferred into a low-calcium dissociation solution containing 0.6 mg/ml papain (Worthington) and 1 mg/ml dithiothreitol (DTT) (Sigma) and incubated for 15 min at 37°C. The femoral arterial segments were washed twice with fresh low-calcium dissociation solution and transferred into a 1 ml vial containing 0.5 mg/ml collagenase (Sigma) and 0.5 mg trypsin inhibitor (Sigma) and incubated at 37°C. Supernatant fractions were collected every 5 minutes and diluted to 1 ml with fresh low-calcium dissociation solution and checked for the appearance of dispersed cells under a microscope. The procedure was repeated by incubating the remaining femoral arteries with 1 ml fresh low-calcium dissociation solution. The collected femoral arterial cell suspension was placed on ice and used for patch-clamp experiments within 6 hours.

### BK_ca_ channel current recordings

Single-channel K^+^ currents were recorded at room temperature from cell-attached and excised inside-out membrane patches of freshly isolated mouse femoral arterial muscle cells using the patch-clamp technique. Channel current recording pipettes were fabricated from borosilicate glass pulled on a 2-stage micropipette puller (PC-84) and heat-polished under a microscope (Narishige MF-83 heat polisher). The recording pipettes were mounted on a three-way hydraulic micromanipulator (Narishige, Tokyo, Japan) for placement of the tips on the cell membrane. High resistance seals (greater than 2–3 giga ohm) were established by applying a slight suction between fire-polished pipette (with tip resistance values between 3-l0 megohm) and cell membranes. The offset potentials between pipette and bath solution were corrected with an offset circuit before each experiment. Pipette potential was clamped, and single-channel currents were recorded using Axopatch 200B amplifier and Digidata 1440A digitizer (Molecular Devices, Sunnyvale, CA) at a 50-kHz sampling rate and filtered online at 5 kHz using a low-pass Bessel filter. Single-channel currents were analyzed using a pClamp software package (Molecular Devices, Sunnyvale CA, USA, pClamp version 10.3) to determine event frequency and open state probability. Slope conductance was determined by fitting the unitary current-voltage relation using least-square linear regression. The effects of NIR light on single-channel BKCa single-channel activity was recorded from 1 to 2 independent replicates of cell-attached or excised inside-out membrane patches obtained from femoral arteries isolated from one mice and mean values per mice (animal) were used to obtain a data point.

### Patch-clamp solutions

Pipette solutions for both cell-attached and excised inside-out patches contained (in mM): KCl 145, CaCl_2_ 1.8, MgCl_2_ 1.1, HEPES 5, with the final pH adjusted to 7.2 with KOH. During recording from either cell-attached or inside-out membrane patches the bath solution was composed of (in mmol/L): KCl 145, CaCl_2_ 1.8, MgCl_2_ 1.1, HEPES 5, ethylene-glycol-bis (β-aminoethyl ether)-N, N, N’, N’-tetraacetic acid (EGTA) 10, with pH adjusted to 7.2 with KOH. This resulted in a calculated final bath [Ca^2+^] of 10^−7^ M. The bath was contained in a volume of 1 ml that was continually exchanged with fresh solution at a rate of 2 ml/min by gravitational flow. To study the calcium sensitivity of single-channel K^+^ currents recorded from inside-out patches of mouse femoral arterial muscle cells the [Ca^2+^] in the recording bath solution was increased from 1 μM to 3 μM.

### BKca knockout mice

A breeding pair of heterozygous Slo ^-/+^ mice developed by AL Meredith were obtained from the lab of Harpreet Singh (Drexel University College of Medicine) [18].

### PCR genotyping of Slo/Mice

Tail snips were digested overnight at 55°C in 750 μl of SNET (20 mM Tris, pH 8, 1mM EDTA, 1% SDS, 0.4 M NaCl) plus 15 μl of 20 mg/ml proteinase K and extracted with an equal volume 1:1 phenol/chloroform. DNA was ethanol-precipitated and 500 ng of DNA (or 2 μl of supernatant) was used in PCR reactions (Promega, Madison WI, supplier’s reaction condition plus 2% DMSO); amplification conditions: 94°C, 2 min; (94°C, 30 s; 55–50°C, 30s; 72°C, 2 min) x 5 cycles; (94°C, 30 s; 50°C, 30 s; 72°C, 2 min) x 30 cycles; 72°C, 5’;4°C, hold). Neo 5’ (5’-ATA GCC TGA AGA ACG AGA TCA GC-3’) and RA 14025 3’ (5’-CCT CAA GAA GGG GAC TCT AAA C-3’) amplify the Slo ^-/-^ allele product of 800 bp. Exon 1 5’-3 (5’-TTC ATC TTG CTC TGG CGGACG-3’) and WT 3’-2 (5’-CCA TAG TCA CCA ATA GCC C-3’) amplify the wild-type product of 332 bp.

### Statistics

All values are expressed as mean Standard Error of the Mean (SEM). Comparisons were made using a Student’s t-test, with the exception of a one-way ANOVA with Holm-Sidak post hoc analysis for vasodilation studies performed over time. Values for p<0.05 were considered significant.

## Results

### R/NIR stimulates vasodilation and requires functional potassium channels

**Fig 1:**
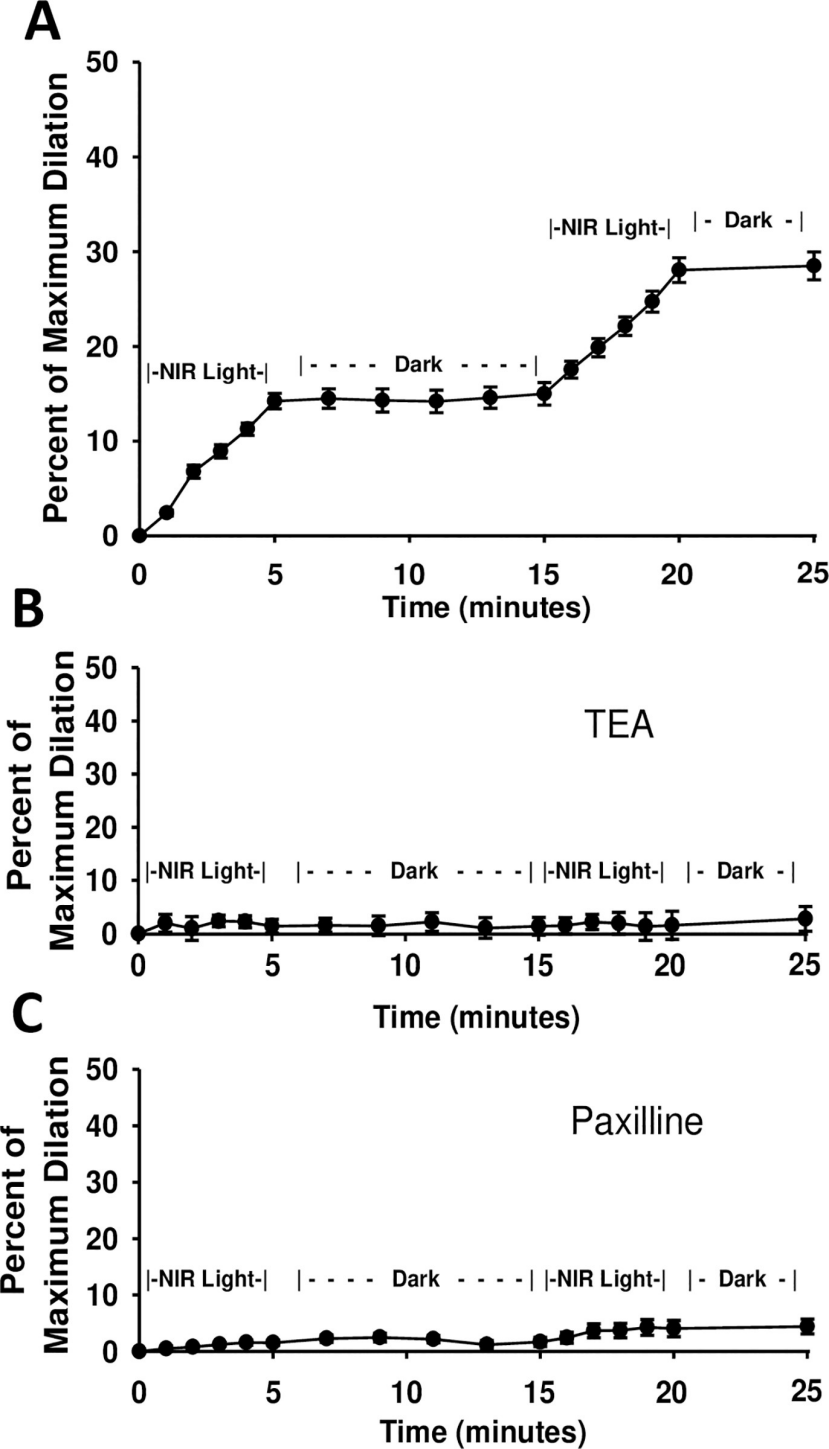
R/NIR stimulates vasodilation and is mediated by potassium channels. Facial arteries from C57BI/6 mice were isolated and pressurized to 60mmHg before pre-constriction with U46619. **(A-C)**. Vessels were exposed to 5 minutes of 670nm NIR Light, 10 minutes in darkness with no light, and then a second exposure of NIR light. (**B).** Preincubation of facial arteries with TEA (1mM for 10min) resulted in no increase in diameter upon energy exposure (R/NIR). (**C).** Preincubation of facial arteries with Paxilline (200nM for 15min) resulted in no increase in vessel diameter upon energy exposure (R/NIR). N = 8 vessels from 4 mice in each group.

R/NIR stimulated vasodilation was previously tested with the mouse facial artery, a surrogate for microvascular function. Consistent with previous observations, there was a 14.2% (±0.81) increase in vessel diameter after 5 min of light exposure to pre-constricted facial arteries (**Fig 1A**) [16,17]. Since potassium channels are key mediators of vasodilation, we tested the impact of channel inhibition on NIR vasodilation. Tetraethylammonium (TEA), a selective voltage gated potassium channel inhibitor, abolished light mediated vasodilation (1.4 ±1.31)) (**Fig 1B**). This inhibitory effect was also observed when paxilline, a specific inhibitor of the large conductance potassium channel (B_Kca_) was administered prior to light treatment (1.6 ±0.47) (**Fig 1C**).

### Electromagnetic energy increases voltage dependent K^+^ channel current activity

**Fig 2:**
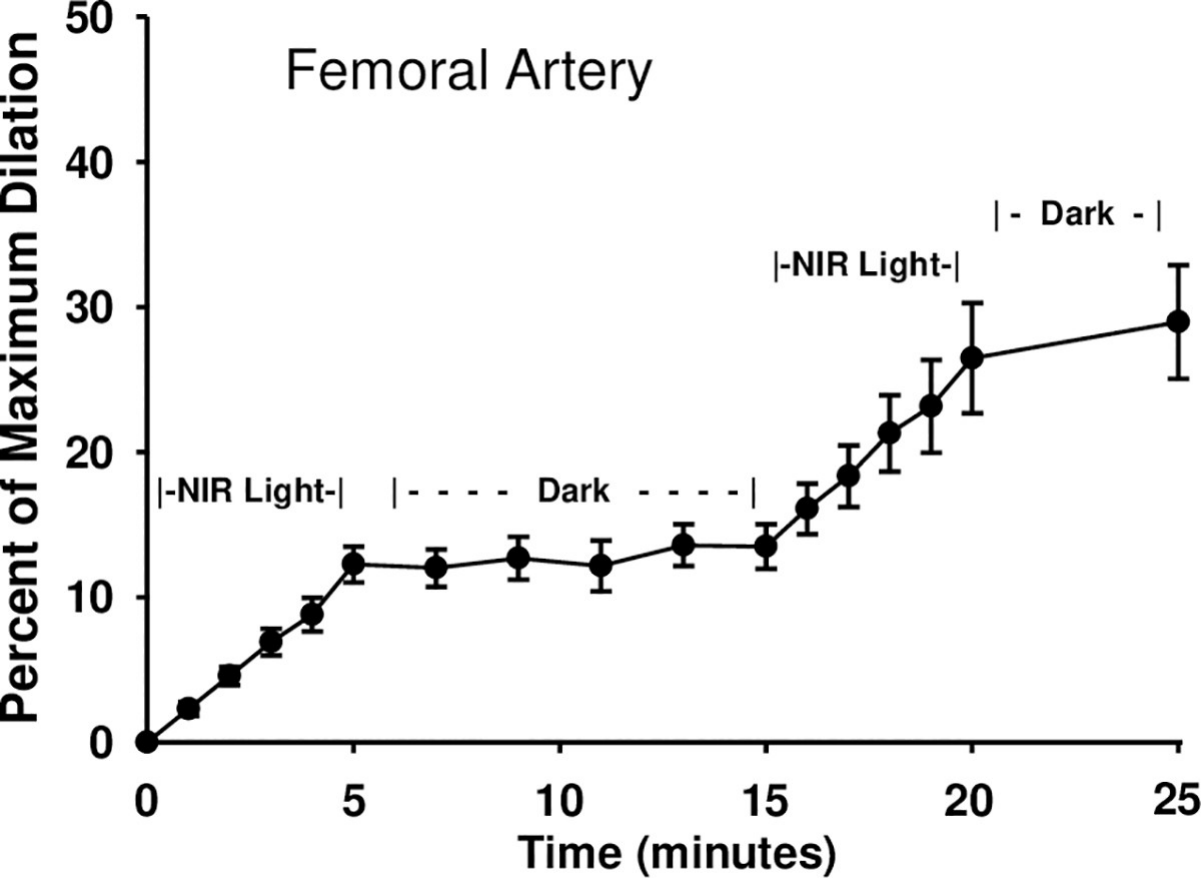
R/NIR vasodilates femoral arteries. Femoral arteries from C57BI/6 mice (n = 8 vessels from 4 mice) were isolated and pressurized to 60mmHg before pre-constriction with U46619. Vessels were exposed to 5 minutes of 670nm NIR Light, 10 minutes in darkness with no light, and then a second exposure of NIR light.

**Fig 3:**
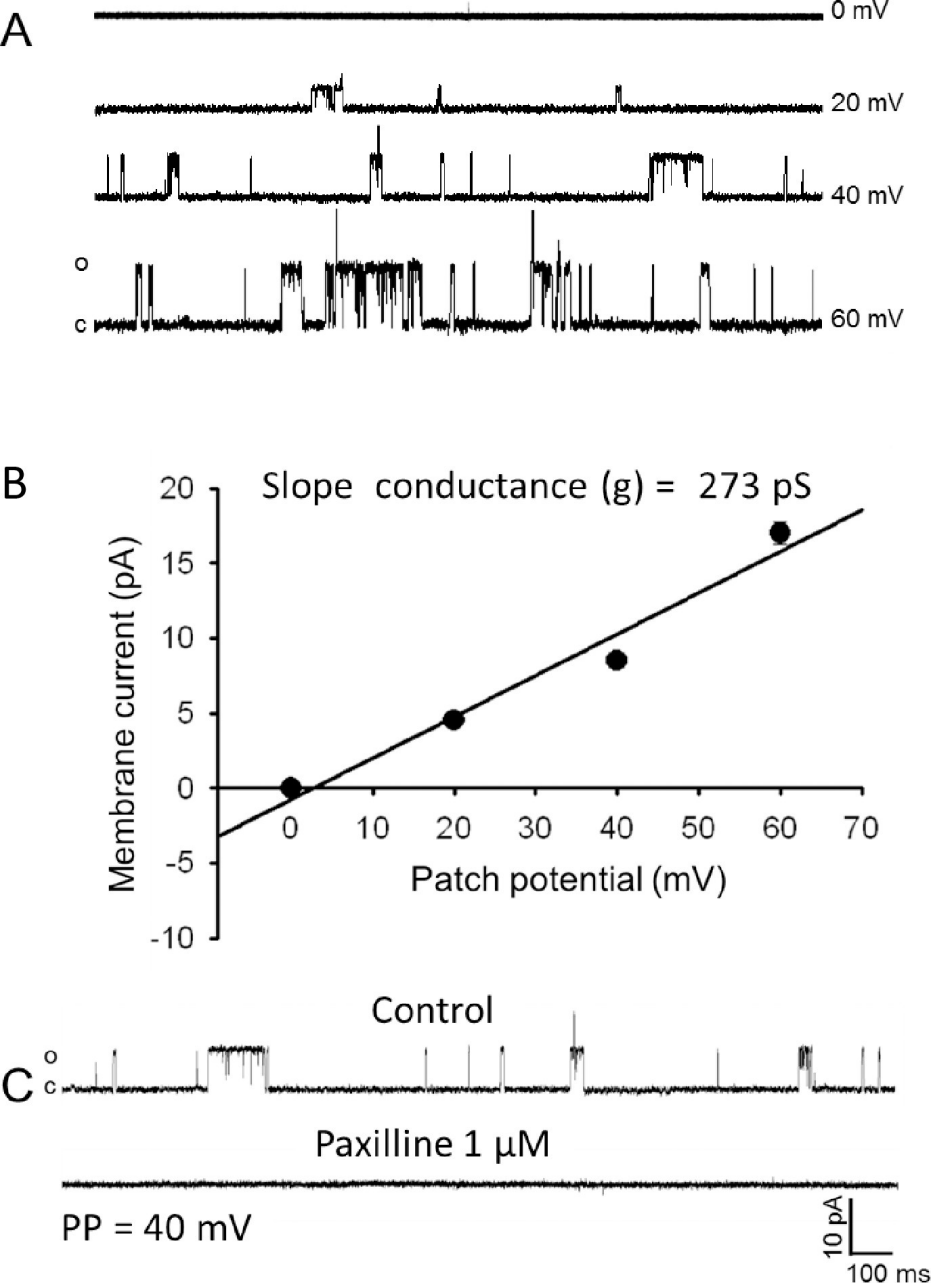
Femoral smooth muscle cells express an active BK_Ca_ channel. Excised inside-out membrane patches of isolated mice femoral arterial smooth muscle cells express a voltage-dependent openings of single-channel K^+^ current (A) with a unitary slope conductance of 273 pS (B) and is sensitive to inhibition by the specific BK_Ca_ channel current inhibitor Paxilline (C).

**Fig 4:**
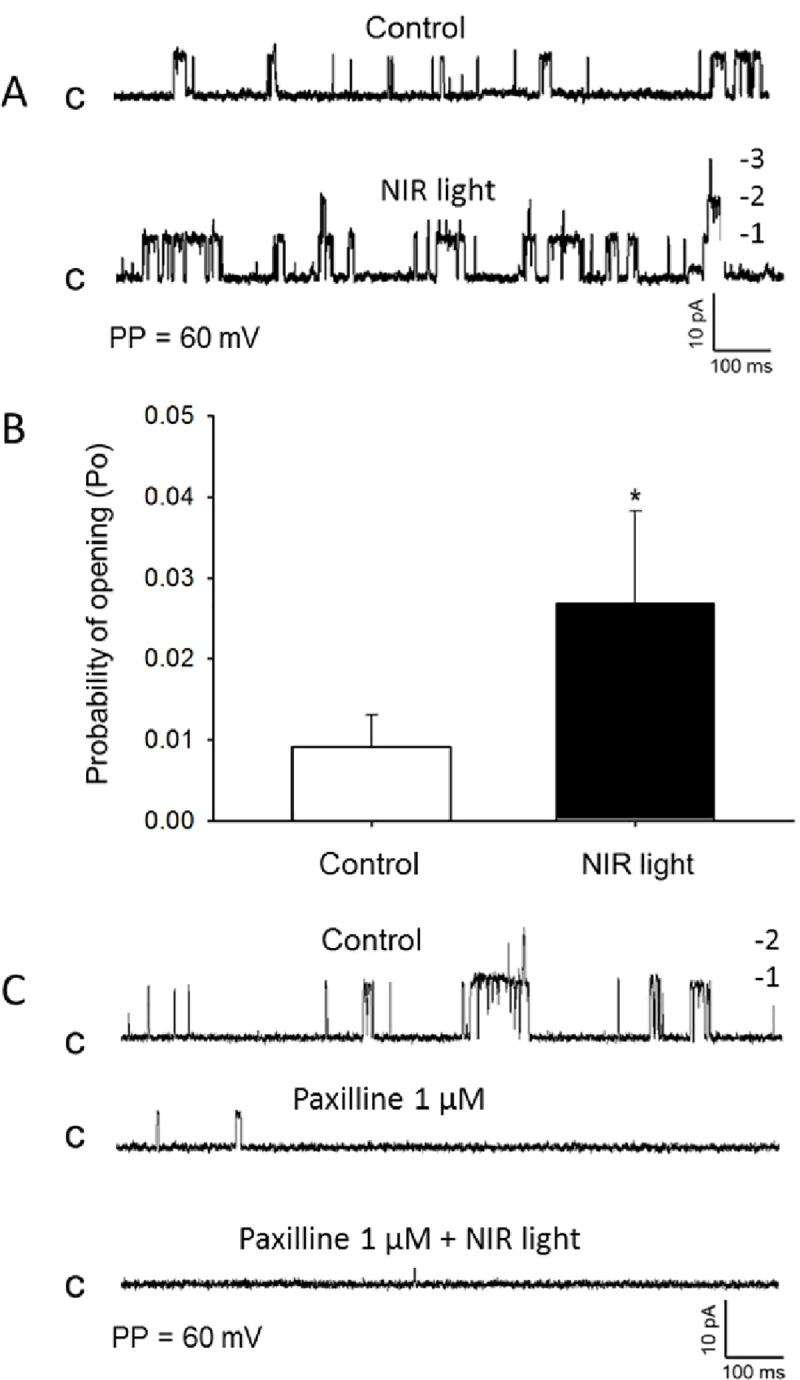
R/NIR increases the opening frequency and open state probability of the BK_Ca_ channel. Exposure to NIR light induced marked increase in opening frequency and probability of opening (Po) (A and B) of a 273 pS BK_Ca_ single-channel current recorded from cell attached patches of wild type mice isolated femoral arterial smooth muscle cells at a patch potential of +40 mV using symmetrical 145 mM KCl recording solution (n = 20 cells from 7 mice, *p< 0.05). Pretreatment of the cell-attached patches with the specific BK_Ca_ channel current inhibitor paxilline (1 μM) prevented the NIR induced increase in the opening frequency of the BKCa single-channel currents (C).

Since increased potassium channel activity is a key mediator of vasodilation, we measured the effect of electromagnetic energy on channel function. Since the facial artery could not yield sufficient dissociated smooth muscle cells, the femoral artery smooth muscle was utilized as a source of cells for patch clamping. To ensure consistency in the physiological responses and the patch clamping current analysis, the response of the femoral artery to R/NIR was tested by pressure myography. As expected, light exposure to the femoral artery elicited a 12.3% change after 5 min (±1.24) (**Fig 2**). The effect plateaued when light was removed and then increased upon re-stimulation with light. There was no significant difference in the magnitude of R/NIR vasodilation in femoral vessels when compared to the facial artery in Fig 1 or previously published observations [16,17]. Patch clamp K^+^ channel current recording from freshly dissociated mouse femoral arterial muscle cells using symmetrical K^+^ (145 mM) recording solutions revealed existence of a 273 pS single K^+^ channel current that displayed voltage-dependent increased openings and sensitivity to inhibition by the selective large conductance Ca^2+^-activated K^+^ channel (BK_Ca_) antagonist paxilline (1 μM) (**Fig 3A–3C**). Exposure to R/NIR induced a significant increase in the opening frequency and open state probability (NPo) of this 273 pS single–channel BK_Ca_ channel current (**Fig 4A and 4B**) indicating that R/NIR light has the capacity to activate the channel. This activation of BKCa channel by R/NIR was completely attenuated following treatment of the mouse femoral arterial muscle cells with the specific BK_Ca_ channel inhibitor paxilline (**Fig 4C**, 1μM) evidence that this channel is the putative mediator of R/NIR induced femoral arterial vasodilation.

**Fig 5:**
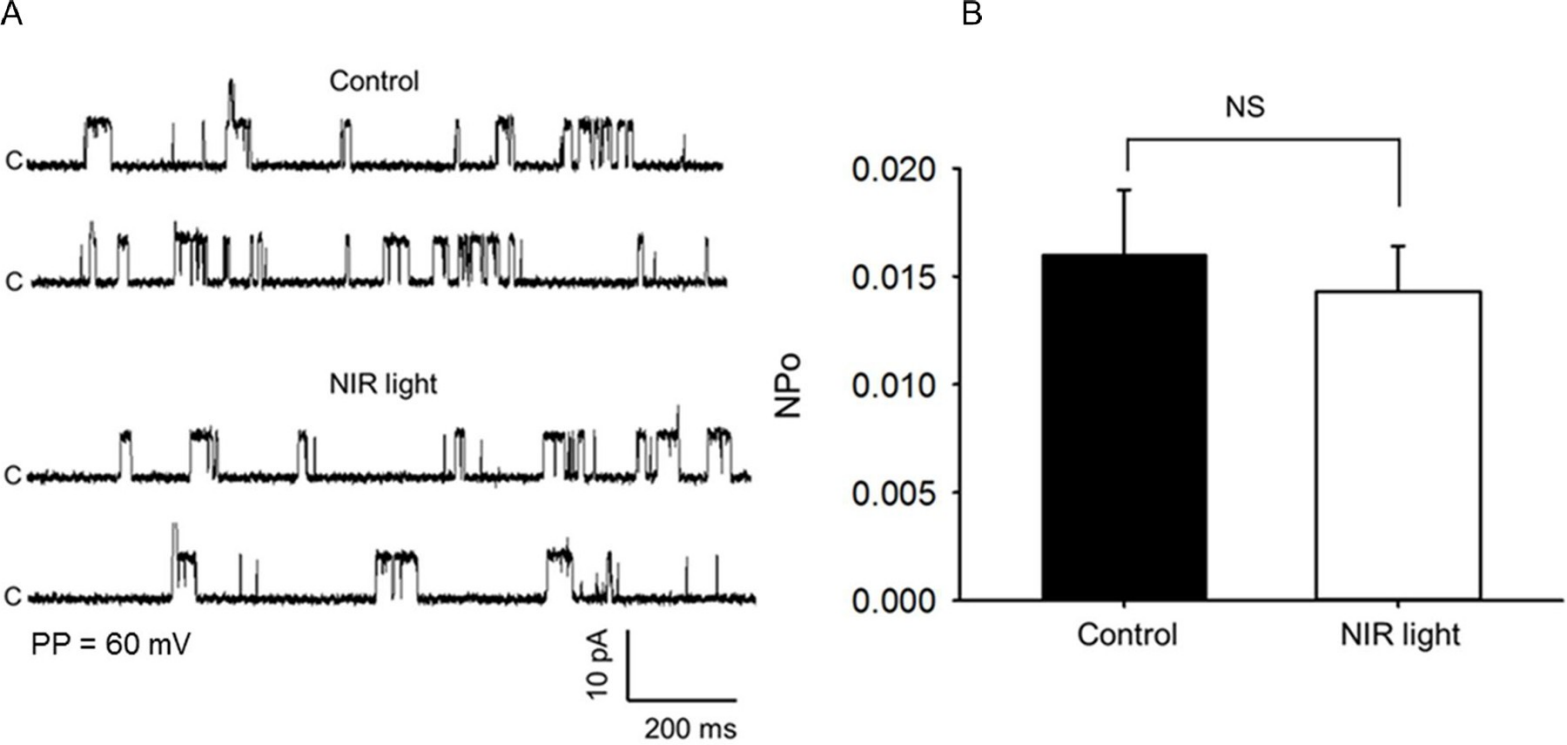
R/NIR does not directly activate the BK_Ca_ channel. Exposure to NIR light has no significant effect (NS, n = 6 animals/group 1–2 independent replicates per animal) on the opening frequency (A) and NPo (B) of the 273 pS BK_Ca_ single channel current recorded from excised inside-out patches of wild type mice isolated femoral arterial smooth muscle cells.

To characterize light’s actions on the regulation of BK_Ca_ channel activity, single channel currents were recorded in excised inside-out membrane patches of wild type femoral arterial smooth muscle cells and the effects of exposure to R/NIR light was examined. As depicted in **Fig 5A and 5B**, no significant increase in the opening frequency and the open state probability (NPo) of the 273 pS BK_Ca_ single-channel current was observed in response to exposure to 670 nm light. These findings reveal that the mechanism of action of light on the BK_Ca_ activity is not a direct modulatory effect on the opening of the channel.

**Fig 6:**
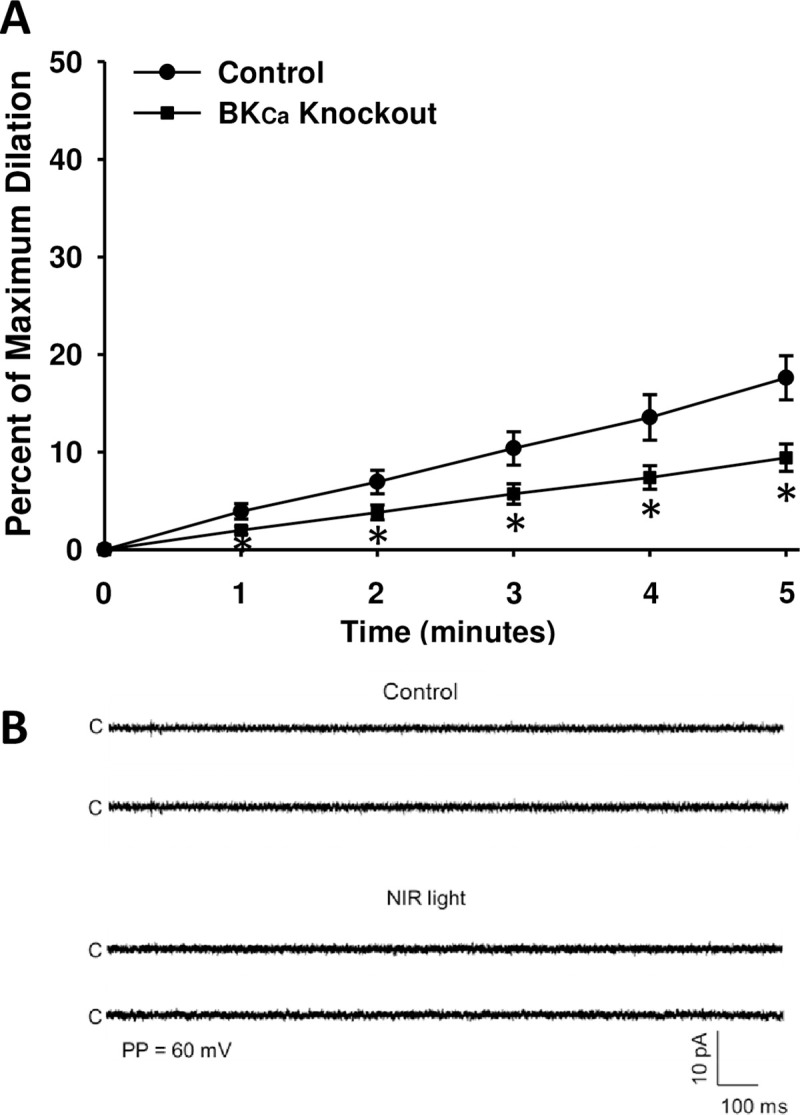
BK_Ca_ Slo-1 knockout mice exhibit loss of channel conductance and impaired R/NIR vasodilation. (**A).** Facial arteries from C57BI/6 and BK_Ca_ Knockout mice were isolated and pressurized to 60mmHg before pre-constriction with U46619. Vessels were then exposed to 670nm NIR light for 5 minutes. There was a significant decrease in R/NIR light mediated dilation in the BK_Ca_ Knockout vessels vs. C57Bl/6 control vessels (*p<0.05) n = 8 vessels from 4 mice in each group **(B)**. No BK_Ca_ single-channel current openings were detected in either under control condition or in response to exposure to R/NIR light during recording from the cell-attached patches of isolated femoral arterial muscle cells of the BK_Ca_ channel knockout mice by employing identical patch clamp recording conditions.

To further confirm the contribution of BK_Ca_ channel activity on light stimulated vasodilation, pressure myograph studies were performed on isolated femoral and facial arterial segments of wild type and BK_Ca_ Slo-1 knockout mice (**Fig 6A**). Exposure to R/NIR light, resulted in a predictable increase in vessel diameter after 5 min in the control group. However, the response to light treatment was blunted in the BK_Ca_ Slo-1knockout (9.4% SEM 1.4, p<0.05), when compared with the wild-type genetic control (17.6% ±2.25) suggesting the BK_Ca_ channel was a significant contributor to R/NIR light induced vasodilation. Patch clamp recordings of single-channel K^+^ currents in SMC isolated from BK_Ca_ Slo-1 knockout demonstrated the loss of light stimulated activation of the 273 pS single–channel BK_Ca_ channel current and supported the observed blunted R/NIR light induced vasodilation response in the arterial segments isolated from BK_Ca_ Slo-1knockout mice (**Fig 6B**). Functional loss of the BK_Ca_ current was confirmed by examining the vasodilatory actions of the specific BK_Ca_ channel activator BMS 191011. Treatment with BMS 191011 (3 μM) evoked increased openings of BK_Ca_ single-channel currents recorded from wild type cell-attached patches but had no effect on those recoded from cell-attached patches of the BK_Ca_ Slo-1 KO mice isolated femoral arterial muscle cells (**S1A and S1B Fig**). Although the effect of light was not completely abolished in the knock-out vessels, there was residual dilation in the BMS 191011 stimulated control (**S1C and S1D Fig**). These results suggest genetic deletion of BK_Ca_ Slo-1 KO can significantly attenuate light induced vasodilation.

## Discussion

This study convincingly demonstrates that 1) electromagnetic energy (R/NIR light, 670 nm) can significantly dilate the facial and femoral arteries 2) this dilation can be abolished by the inhibition of the large conductance potassium channels with paxilline and low concentration of TEA, 3) Exposure to R/NIR light increases the opening frequency and open state probability (NPo) of a 273 pS BK_Ca_ single-channel current in wild type mice but not in BK_Ca_ Slo-1 KO mice isolated SMC, 4) The mechanism of action for electromagnetic energy (R/NIR light) to cause dilation requires functional BK_Ca_ channel as the induced vasodilation is eliminated pharmacologically using the specific BKCa channel inhibitor paxilline and by genetic knockout of BK_Ca_ Slo-1. 5) There is no direct action of light in modulating BK_Ca_ channel current activity in the murine arterial SMC.

The action of light energy to modulate ion channel function remains relatively novel. In algae, channelrhodopsins 1 and 2 are established light gated cation channels which are activated at wavelengths in the blue and green spectrum (436 nm to 587nm) [19]. Photobiomodulation, the application of low energy visible and near infrared light to tissues, has been attributed to modulation of the TrpV1 and TrpV4 channels, which are non-selective cation channels responsible for modulating neural responses to pain, pressure, and heat [20–22]. However, in whole cell patch recordings in trp1 expressing oocytes, the activation of the TrpV1 channel by red light was minimal when compared to green wavelength [23]. To our knowledge there is no published evidence describing light as a regulator of BKCa channel function. TrpV4 indirectly activates BKCa channel function by increasing calcium conductance, however TrpV4 activation is limited to light in the blue spectrum [24,25]. The present study, although limited to light in the far-red spectrum, demonstrates robust activation of the BK_Ca_ channel current.

Vascular reactivity depends on the regulation of smooth muscle contractility through a complex interplay between intracellular potassium and calcium handling. Rising intracellular calcium levels trigger compensatory efflux of K^+^ ions which act as a negative feedback mechanism through repolarization. The large conductance potassium channel (BK_Ca_) is the major contributor to re-establishing membrane potential and hyperpolarizing the smooth muscle cell [26]. Its structure consists of an alpha subunit containing 7 transmembrane domains attributed to voltage sensing and ion pore formation and a regulatory beta subunit has two transmembrane domains. The BK_Ca_ channel is a major end effector of vascular reactivity. Factors which contribute to its modulation include intracellular calcium concentration, membrane potential, and phosphorylation.

Nitric oxide is a well described regulator of BKCa channel activity through both direct and indirect mechanisms [27–29]. Direct activation of the channel by light appears to be unlikely, since exposure to R/NIR light failed to modulate the open state probability (NPo) of BK_Ca_ single-channel current when recorded from excised inside-out patches of mice. Therefore, the R/NIR light induced modulation of BK_Ca_ is likely to be indirect and possibly mediated by increases in intracellular NO. The NO/cGMP/PKG regulation of BKCa mediated vasodilation is well described [29]. NO binding to the soluble guanylate cyclase leads to activation of PKG-I and phosphorylation of the channel. Since light treatment increases the open state probability in cell attached patches, the indirect activation of the channel by endogenous production or release of NO is a candidate mechanism of action. This hypothesis will be the focus of future investigation.

Although significant cellular NO production is through the enzymatic oxidation of L-arginine to L-citrulline and NO, there is increasing evidence that NO is present in other chemical forms (e.g., nitrite, iron nitrosyl globin compounds, S-nitrosothiols and di nitrosyl iron compounds) [17,30–34]. There is growing evidence that R/NIR increases NO levels through its action on these compounds. Previously we identified the action of light on S-nitrosothiol compounds, a potential NO source [17]. We have also described the ability of light to release NO from nitrosyl-hemoglobin and nitrosyl-myoglobin in the blood and cardiac tissue [14]. These are likely candidate pools for generating intracellular NO to activate the cGMP/PKG pathway to activate the BKCa channel.

There are some limitations to the current study which require discussion. The complete cellular mechanism for R/NIR activation of the BK_Ca_ channel necessitates further study and our current results suggest indirect action of light. Further experiments will be required to dissect the intracellular mediators activated by R/NIR light to alter channel function. Moreover, the relative significance of light stimulated SMC activation in the larger context of vascular function needs further study. Our previous results using the same vessel type indicates R/NIR induced vasodilation is largely endothelial dependent, yet clearly the BKCa channel is activated in the absence of the endothelial cell further highlighting the need for further investigation [16]. One potential explanation for these differences may exist in the relative abundance of the mediator within endothelial and smooth muscle cells. Light exposure to intact vessels results in exocytosis of S-nitrosothiols into the bath and vasodilation in a cGMP dependent manner [16,35]. The effects of SMC channel activation by red light can be detected in patch clamp recording but appear less significant when compared to the endothelial derived NO source. It is important to note both the smooth muscle and endothelial pathways of vasodilation share the BKCa channel as an end effector and therefore can be inhibited by paxilline. We have purposefully kept light intensity and wavelength standardized to ensure proper comparisons between the endothelial cell and the smooth muscle cell responses to R/NIR light. A final limitation is the observed residual vasodilation in knock out animals, despite the absence of BK_Ca_ channel activity, could be regarded to reflect compensation by other ion channels to maintain survival.

In summary, 670 nm light can stimulate arterial vasodilation and is markedly reduced by pharmacological inhibition of the BK_Ca_ channel. Exposure to R/NIR light increases the open state probability of the BK_Ca_ channel, and this increase is abolished with genetic knockout and by specific pharmacological inhibition of the channel. Exposure to light does not directly modulate the BK_Ca_ channel current activity, as demonstrated by the lack of effect during recoding from excised inside-out membrane patches. These findings are exciting as they show that R/NIR light energy stimuli can regulate BK_Ca_ channel activity in arterial muscle cells and assist in our understanding of applying R/NIR light to noninvasive therapies for wound healing and impaired vascular function.

## Supporting information

S1 Fig Treatment with the specific BK_Ca_ channel activator BMS 191011 markedly increased the opening frequencies of BK_Ca_ single-channel current recorded from cell-attached patches of wild type (A) compared to control, whereas there was no detectable BK_Ca_ single- channel current activity under control condition and following treatment with the BK_Ca_ single-channel activator BMS 191011 during recording from cell-attached patches of BK_Ca_ KO mice isolated femoral arterial muscle cells (B). Cannulated and pressurized wild type mice femoral arterial segments had a greater dilation in response to treatment with BMS 191011 as compared to that similarly determined in the BK_Ca_ KO mice isolated femoral arterial segments over a period of 10 min (C, D n = 8 vessels from 4 mice). *p<0.05.
(TIF)

## References

[1] Vita JA, Hamburg NM. Does endothelial dysfunction contribute to the clinical status of patients with peripheral arterial disease? Canadian Journal of Cardiology. 2010;26:45A–50A. doi: 10.1016/s0828-282x(10)71062-x 20386761

[2] Willey J, Mentias A, Vaughan-Sarrazin M, McCoy K, Rosenthal G, Girotra S. Epidemiology of lower extremity peripheral artery disease in veterans. Journal of vascular surgery. 2018. doi: 10.1016/j.jvs.2017.11.083 29588132 PMC6132057

[3] Peacock JM, Keo HH, Duval S, Baumgartner I, Oldenburg NC, Jaff MR, et al. The incidence and health economic burden of ischemic amputation in Minnesota, 2005–2008. Preventing chronic disease. 2011;8(6):A141. 22005634 PMC3221580

[4] Kalbaugh CA, Loehr L, Wruck L, Lund JL, Matsushita K, Bengtson LG, et al. Frequency of Care and Mortality Following an Incident Diagnosis of Peripheral Artery Disease in the Inpatient or Outpatient Setting: The ARIC (Atherosclerosis Risk in Communities) Study. Journal of the American Heart Association. 2018;7(8):e007332. doi: 10.1161/JAHA.117.007332 29654201 PMC6015432

[5] Mahoney EM, Wang K, Keo HH, Duval S, Smolderen KG, Cohen DJ, et al. Vascular hospitalization rates and costs in patients with peripheral artery disease in the United States. CirculationCardiovascular quality and outcomes. 2010;3(6):642–51. doi: 10.1161/CIRCOUTCOMES.109.930735 20940249

[6] Neupane S, Edla S, Maidona E, Sweet MC, Szpunar S, Davis T, et al. Long‐term outcomes of patients with diabetes mellitus undergoing percutaneous intervention for popliteal and infrapopliteal peripheral arterial disease. Catheterization and Cardiovascular Interventions. 2018;92(1):117–23. doi: 10.1002/ccd.27571 29536612

[7] Caetano KS, Frade MA, Minatel DG, Santana LA, Enwemeka CS. Phototherapy improves healing of chronic venous ulcers. Photomedicine and laser surgery. 2009;27(1):111–8. doi: 10.1089/pho.2008.2398 19196110

[8] Desmet KD, Paz DA, Corry JJ, Eells JT, Wong-Riley MT, Henry MM, et al. Clinical and experimental applications of NIR-LED photobiomodulation. Photomedicine and laser surgery. 2006;24(2):121–8. doi: 10.1089/pho.2006.24.121 16706690

[9] Erdle BJ, Brouxhon S, Kaplan M, Vanbuskirk J, Pentland AP. Effects of continuous‐wave (670‐nm) red light on wound healing. Dermatologic Surgery. 2008;34(3):320–5. doi: 10.1111/j.1524-4725.2007.34065.x 18177400

[10] Minatel DG, Enwemeka CS, Franca SC, Frade MA. Phototherapy (LEDs 660/890nm) in the treatment of leg ulcers in diabetic patients: case study. Anais Brasileiros de Dermatologia. 2009;84(3):279–83. doi: 10.1590/s0365-05962009000300011 19668943

[11] Maegawa Y, Itoh T, Hosokawa T, Yaegashi K, Nishi M. Effects of near-infrared low-level laser irradiation on microcirculation. Lasers in surgery and medicine. 2000;27(5):427–37. 2-A. doi: 10.1002/1096-9101(2000)27:5&lt;427::AID-LSM1004&gt;3.0.CO;2-A 11126437

[12] Plass CA, Loew HG, Podesser BK, Prusa AM. Light-induced vasodilation of coronary arteries and its possible clinical implication. The Annals of Thoracic Surgery. 2012;93(4):1181–6. doi: 10.1016/j.athoracsur.2011.12.062 22381453

[13] Batenburg WW, Kappers MH, Eikmann MJ, Ramzan SN, de Vries R, Danser AH. Light-induced vs. bradykinin-induced relaxation of coronary arteries: do S-nitrosothiols act as endothelium-derived hyperpolarizing factors? J Hypertens. 2009;27(8):1631–40. Epub 2009/05/08. doi: 10.1097/HJH.0b013e32832bff54 . 19421072

[14] Lohr NL, Keszler A, Pratt P, Bienengraber M, Warltier DC, Hogg N. Enhancement of nitric oxide release from nitrosyl hemoglobin and nitrosyl myoglobin by red/near infrared radiation: potential role in cardioprotection. Journal of Molecular and Cellular Cardiology. 2009;47(2):256–63. doi: 10.1016/j.yjmcc.2009.03.009 19328206 PMC4329292

[15] Lohr NL, Ninomiya JT, Warltier DC, Weihrauch D. Far Red/Near Infrared Light Treatment Promotes Femoral Artery Collateralization in the Ischemic Hindlimb. Journal of Molecular and Cellular Cardiology. 2013. doi: 10.1016/j.yjmcc.2013.05.007 23702287 PMC3747970

[16] Keszler A, Lindemer B, Weihrauch D, Jones D, Hogg N, Lohr NL. Red/near infrared light stimulates release of an endothelium dependent vasodilator and rescues vascular dysfunction in a diabetes model. Free Radical Biology and Medicine. 2017;113:157–64. doi: 10.1016/j.freeradbiomed.2017.09.012 28935419 PMC5699925

[17] Keszler A, Lindemer B, Hogg N, Lohr NL. Ascorbate attenuates red light mediated vasodilation: Potential role of S-nitrosothiols. Redox Biol. 2019;20:13–8. Epub 2018/09/28. doi: 10.1016/j.redox.2018.09.008 ; PubMed Central PMCID: PMC6156744. 30261342 PMC6156744

[18] Meredith AL, Thorneloe KS, Werner ME, Nelson MT, Aldrich RW. Overactive bladder and incontinence in the absence of the BK large conductance Ca2+-activated K+ channel. J Biol Chem. 2004;279(35):36746–52. Epub 2004/06/09. doi: 10.1074/jbc.M405621200 . 15184377

[19] Wang Y, Huang YY, Wang Y, Lyu P, Hamblin MR. Photobiomodulation (blue and green light) encourages osteoblastic-differentiation of human adipose-derived stem cells: role of intracellular calcium and light-gated ion channels. Sci Rep. 2016;6:33719. Epub 2016/09/22. doi: 10.1038/srep33719; PubMed Central PMCID: PMC5030629. 27650508 PMC5030629

[20] Kim C. Transient receptor potential ion channels and animal sensation: lessons from Drosophila functional research. J Biochem Mol Biol. 2004;37(1):114–21. Epub 2004/02/06. doi: 10.5483/bmbrep.2004.37.1.114 . 14761309

[21] Nisembaum LG, Besseau L, Paulin C-H, Charpantier A, Martin P, Magnanou E, et al. In the heat of the night: thermo-TRPV channels in the salmonid pineal photoreceptors and modulation of melatonin secretion. Endocrinology. 2015;156(12):4629–38. doi: 10.1210/en.2015-1684 26389691

[22] Wang L, Zhang D, Schwarz W. TRPV Channels in Mast Cells as a Target for Low-Level-Laser Therapy. Cells. 2014;3(3):662–73. Epub 2014/06/28. doi: 10.3390/cells3030662 ; PubMed Central PMCID: PMC4197630. 24971848 PMC4197630

[23] Gu Q, Wang L, Huang F, Schwarz W. Stimulation of TRPV1 by green laser light. Evidence-Based Complementary and Alternative Medicine. 2012;2012. doi: 10.1155/2012/857123 23365602 PMC3539758

[24] Earley S, Heppner TJ, Nelson MT, Brayden JE. TRPV4 forms a novel Ca2+ signaling complex with ryanodine receptors and BKCa channels. Circulation research. 2005;97(12):1270–9. doi: 10.1161/01.RES.0000194321.60300.d6 16269659

[25] Villegas D, Giard O, Brochu-Gaudreau K, Rousseau É. Activation of TRPV4 channels leads to a consistent tocolytic effect on human myometrial tissues. European Journal of Obstetrics & Gynecology and Reproductive Biology: X. 2021;10:100124. doi: 10.1016/j.eurox.2021.100124 33733088 PMC7941160

[26] Bentzen BH, Olesen S-P, Rønn LCB, Grunnet M. BK channel activators and their therapeutic perspectives. Frontiers in Physiology. 2014;5(389). doi: 10.3389/fphys.2014.00389 25346695 PMC4191079

[27] Bolotina VM, Najibi S, Palacino JJ, Pagano PJ, Cohen RA. Nitric oxide directly activates calcium-dependent potassium channels in vascular smooth muscle. Nature. 1994;368(6474):850–3. doi: 10.1038/368850a0 7512692

[28] Ishikawa T, Hume JR, Keef KD. Regulation of Ca2+ channels by cAMP and cGMP in vascular smooth muscle cells. Circulation research. 1993;73(6):1128–37. doi: 10.1161/01.res.73.6.1128 8222084

[29] Sausbier M, Schubert R, Voigt V, Hirneiss C, Pfeifer A, Korth M, et al. Mechanisms of NO/cGMP-dependent vasorelaxation. Circulation research. 2000;87(9):825–30. doi: 10.1161/01.res.87.9.825 11055988

[30] Bir SC, Pattillo CB, Pardue S, Kolluru GK, Shen X, Giordano T, et al. Nitrite anion therapy protects against chronic ischemic tissue injury in db/db diabetic mice in a NO/VEGF-dependent manner. Diabetes. 2014;63(1):270–81. doi: 10.2337/db13-0890 24009258 PMC4179307

[31] Cosby K, Partovi KS, Crawford JH, Patel RP, Reiter CD, Martyr S, et al. Nitrite reduction to nitric oxide by deoxyhemoglobin vasodilates the human circulation. Nature medicine. 2003;9(12):1498–505. doi: 10.1038/nm954 14595407

[32] Li H, Hemann C, Abdelghany TM, El-Mahdy MA, Zweier JL. Characterization of the mechanism and magnitude of cytoglobin-mediated nitrite reduction and nitric oxide generation under anaerobic conditions. The Journal of biological chemistry. 2012. doi: 10.1074/jbc.M112.342378 22896706 PMC3476328

[33] Shiva S, Huang Z, Grubina R, Sun J, Ringwood LA, MacArthur PH, et al. Deoxymyoglobin is a nitrite reductase that generates nitric oxide and regulates mitochondrial respiration. Circulation research. 2007;100(5):654–61. doi: 10.1161/01.RES.0000260171.52224.6b 17293481

[34] Keszler A, Diers AR, Ding Z, Hogg N. Thiolate-based dinitrosyl iron complexes: Decomposition and detection and differentiation from S-nitrosothiols. Nitric Oxide. 2017;65:1–9. doi: 10.1016/j.niox.2017.01.007 28111306 PMC5663227

[35] Weihrauch D, Keszler A, Lindemer B, Krolikowski J, Lohr NL. Red light stimulates vasodilation through extracellular vesicle trafficking. Journal of Photochemistry and Photobiology B: Biology. 2021:112212. doi: 10.1016/j.jphotobiol.2021.112212 34049180 PMC8240139

