# Supplementary figures and images for: Electromagnetic energy (670 nm) stimulates vasodilation through activation of the large conductance potassium channel (BKCa)
Source: PLoS One. 2021 Oct 5;16(10):e0257896. doi: 10.1371/journal.pone.0257896 (PMC8491904; doi:10.1371/journal.pone.0257896)

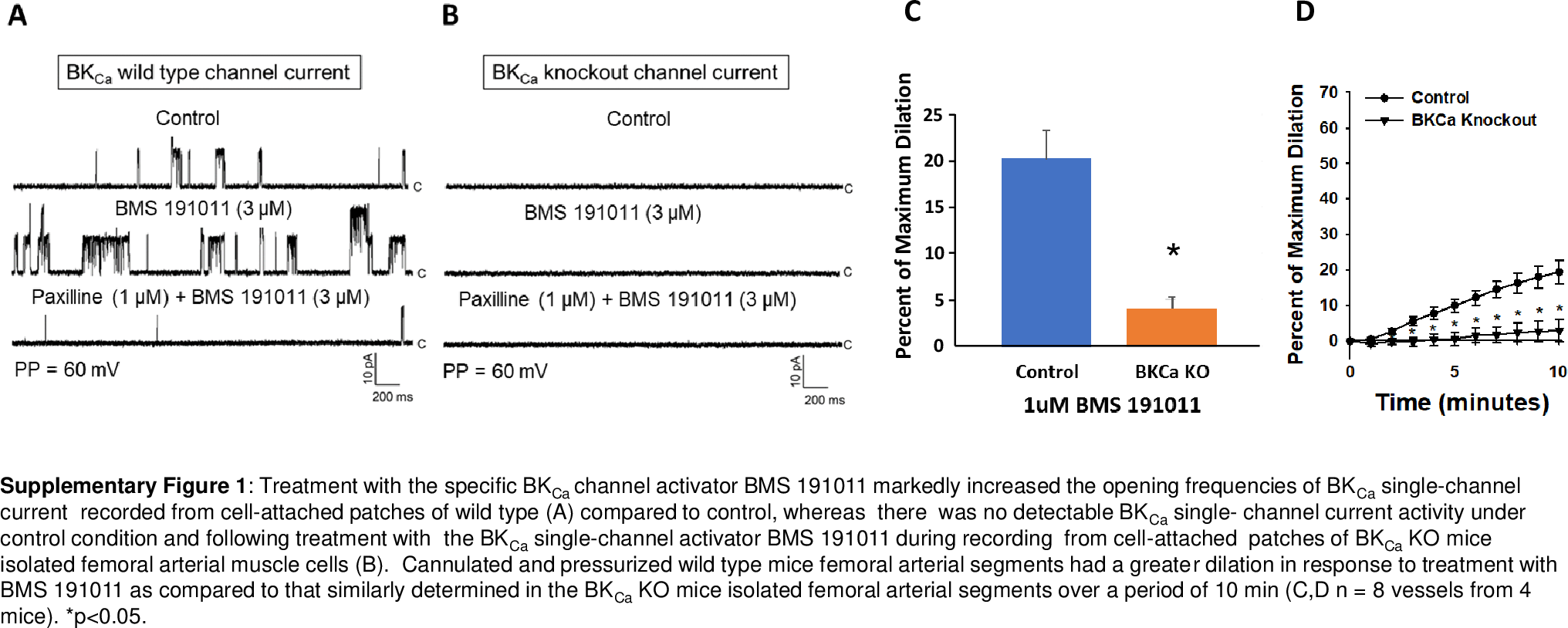

Supplement: S1 Fig — Treatment with the specific BKCa channel activator BMS 191011 markedly increased the opening frequencies of BKCa single-channel current recorded from cell-attached patches of wild type (A) compared to control, whereas there was no detectable BKCa single- channel current activity under control condition and following treatment with the BKCa single-channel activator BMS 191011 during recording from cell-attached patches of BKCa KO mice isolated femoral arterial muscle cells (B). Cannulated and pressurized wild type mice femoral arterial segments had a greater dilation in response to treatment with BMS 191011 as compared to that similarly determined in the BKCa KO mice isolated femoral arterial segments over a period of 10 min (C, D n = 8 vessels from 4 mice). *p<0.05. (TIF) [file pone.0257896.s001.tif]
